# Optical investigations and photoactive solar energy applications of new synthesized Schiff base liquid crystal derivatives

**DOI:** 10.1038/s41598-021-94533-6

**Published:** 2021-07-22

**Authors:** Fowzia S. Alamro, Sobhi M. Gomha, Mohamed Shaban, Abeer S. Altowyan, Tariq Z. Abolibda, Hoda A. Ahmed

**Affiliations:** 1 Department of Chemistry, College of Science, Princess Nourah bint Abdulrahman University, Riyadh, 11671 Saudi Arabia; 2 Department of Chemistry, Faculty of Science, Cairo University, Cairo, 12613 Egypt; 3 Chemistry Department, Faculty of Science, Islamic University in Almadinah Almonawara, Almadinah Almonawara, 42351 Saudi Arabia; 4 Nanophotonics and Applications Labs, Department of Physics, Faculty of Science, Beni-Suef University, Beni-Suef, 62514 Egypt; 5 Department of Physics, Faculty of Science, Islamic University in Almadinah Almonawara, Almadinah Almonawara, 42351 Saudi Arabia; 6 Department of Physics, College of Science, Princess Nourah bint Abdulrahman University, Riyadh, 11671 Saudi Arabia; 7 Chemistry Department, College of Sciences, Taibah University, Yanbu, 30799 Saudi Arabia

**Keywords:** Optical materials and structures, Optical techniques

## Abstract

New homologues series of liquid crystalline materials namely, (*E*)-3-methoxy-4-[(*p*-tolylimino)methyl]phenyl 4-alkloxybenzoates (**I-n**), were designed and evaluated for their mesomorphic and optical behavior. The prepared series constitutes three members that differ from each other by the terminally attached alkoxy chain group, these vary between 6 and 12 carbons. A laterally OCH_3_ group is incorporated into the central benzene ring in meta position with respect to the ester moiety. Mesomorphic characterizations of the prepared derivatives are conducted using differential scanning-calorimetry (DSC), polarized optical-microscopy (POM). Molecular structures were elucidated by elemental analyses and NMR spectroscopy. DSC and POM investigations revealed that all the synthesized derivatives are purely nematogenic exhibiting only nematic (N) mesophase, except for the longest chain derivative (**I-12**) that is dimorphic possesses smectic A and N phases. Moreover, all members of the group have a wide mesomorphic range with high thermal nematic stability. A comparative study was established between the present derivative (**I-6**) and their previously prepared isomer. The results indicated that the location exchange of the polar compact group (CH_3_) influences the N mesophase stability and range. The electrical measurements revealed that all synthesized series **I-n** show Ohmic behaviors with effective electric resistances in the GΩ range. Under white light illumination, the effective electric conductivity for the compound **I-8** is five times that obtained in dark conditions. This derivative also showed two direct optical band gaps in the UV and visible light range. In addition, **I-6 **has band energy gaps of values 1.07 and 2.79 eV, which are suitable for solar energy applications.

## Introduction

Today, the developments of organic solar cells have great progress in the last years^1–9^. For solar energy applications such as catalytic photo-degradation of dyes, solar hydrogen generation, photo-electrochemical water splitting, and solar cells; bandgap engineering and optical property control are critical parameters^10–14^. Low molar mass molecule solar cell also possesses a great potential^15–19^. Organic solar cells are promising for industrial applications because of their possibly low-cost methods. Innovative characteristics of organic solar cells such as lightweight, flexibility, cheap, and solution processability have attracted considerable attention from scientists and technologists. Modern types prove commercial inexpensive with excellent efficiencies^20^.

Recently, liquid crystals (LCs) proved to have wide applications as optical materials; areas of technological applications such as light-emitting diodes, displays, and semi- and photoconductors^21–23^. Numerous documents investigated photovoltaic effects in symmetrical cells filled with LCs^24–26^. Photovoltaic impact analogous to that of some of the better organic solar cells was investigated^24,27,28^.

The development of new geometrical shapes to achieve the desired properties for device applications is one of our interests^29–33^. So that, the choice of the terminal flexible alkoxy/alkyl chains, terminal and lateral polar groups, as well as the mesogenic spacers, are important criteria in the designing of novel LCs for proper characteristic technological applications. In addition, the molecular structure enables some considerable modifications in the mesomorphic behavior and plays an essential role in the observation, type, and thermal stability of the formed mesophase^29–33^.

Generally, the introductions of lateral-substituent will increment the intermolecular separations, which broaden the core moiety and leads to a decrement in the lateral interactions^34–37^. Moreover, as the breadth of the molecule increases, the thermal stability of formed mesophases is reduced^37^. The insertion of a lateral group on the aromatic ring of the mesogenic part influenced the mesophase transition temperature range of the smectic phases and increased spontaneous polarization. The addition of electron-donating groups in the LCs skeletons may be strongly impacting their polarizability and/or polarity as well as their geometric structures. Consequently, it affects the transition phase temperature, kind of mesophase, and other thermal and geometrical parameters essential for better prosperities of the LCs materials^38–41^. The laterally extended Liquid Crystals derivatives have been widely reported for organic electronics applications^42,43^. In another work, the di-laterally substituted LCs were designed and used for organic light‐emitting devices creations^44^. Moreover, the small LCs molecules^45^ were used for the construction and development of all-small-molecule non-fullerene solar cells.

Schiff base and hydrazone derivatives are well known as valuable intermediates in the synthesis of many organic compounds that exhibit a multitude of many applications^46–50^. The Azomethine spacer maintains the rigidity and linearity of the geometrical shape thus enhancing the mesophase thermal stability. In addition, imines are prone to hydrolysis and thermal decomposition^51,52^. The kinetic studies on the hydrolysis of Schiff bases were reported^53^. Optical activity of –CH=N– linking moiety has been investigated^54,55^ due to the photo-efficiency with wavelength-dependent on the chemical compositions of Schiff base-based molecules. Wide low molecular mass azomethine systems and twist bend nematic mesophase have been studies^56–58^. On the other hand, the conjugative interactions between the ester group and the phenyl rings play an important role to enhance the mesomorphic properties. In general, the architecture of an organic compound resulted in a remarkable change in the mesomorphic characteristics depending on its molecular conformation^59^. Moreover, the terminal flexible chains or polar compact substituents have essential roles in the phase transition properties^60^.

The present studies aim to synthesize new Schiff base derivatives of laterally methoxy group, with different terminal alkoxy chain length, namely, (*E*)-3-methoxy-4-[(*p*-tolylimino)methyl]phenyl 4-alkloxybenzoates, **I-n**.

On the phenyl –CH=N– linkage methyl substituent is attached, while on the other terminal phenyl ring different lengths of alkoxy group are attached, and the lateral CH_3_O group is introduced into the central ring. Moreover, the investigation aims to evaluate the effect of the change terminal length of flexible chain on the mesomorphic properties of the prepared series. Furthermore, a comparison is established between the present homologues and the previously corresponding isomers to impact the effect of exchange the location of terminals on the mesomorphic behavior. Furthermore, the study also aims to investigate the electric and optical properties, the electric resistance, conductance, energy gap, as well as Urbach Energy.
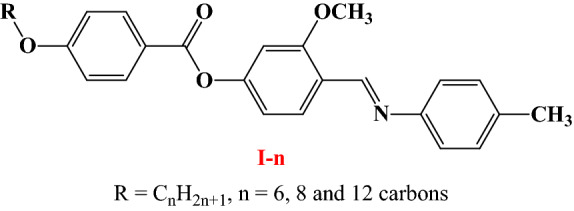


## Experimental

### Synthesis

**Figure 1:**
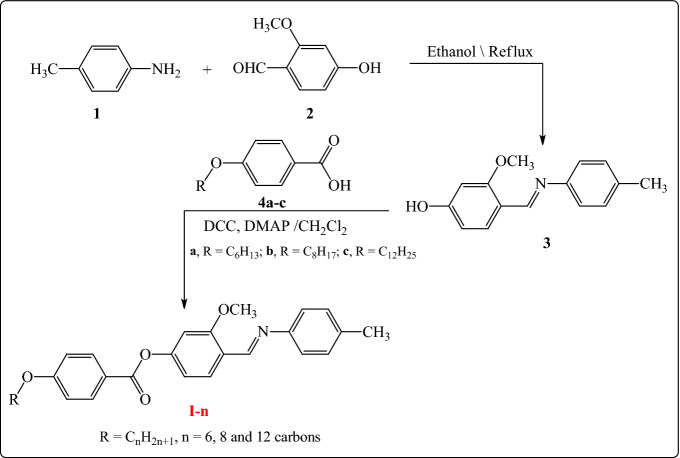
Synthesis route of title compounds **I-n**.

The present homologue **I-n** was prepared as the following Fig. 1:

The physical analyses data of products **I-n** are given in Supplementary Materials.

### Films preparation

A very thin layer of the sample was prepared by sandwiching them between a glass slide and a coverslip.The dimensions of the cell was 22 mm × 22 mm × 0.03 mm.” i.e., the film thickness was ~ 30 μm. The temperature of cell was controlled using temperature controller with an accuracy ± 0.1 °C.

## Results and discussion

### Mesomorphic and optical investigations of present derivatives, I-n

**Figure 2:**
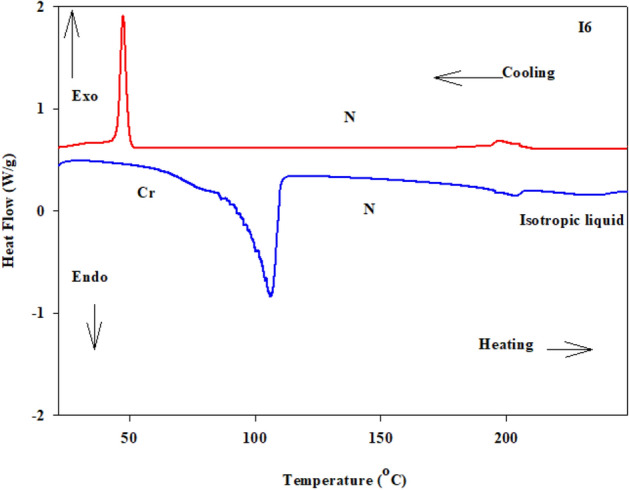
DSC thermograms of compounds **I-6** at a rate of ± 10 °C/min (**a**) recorded from heating and cooling scans.

**Figure 3:**
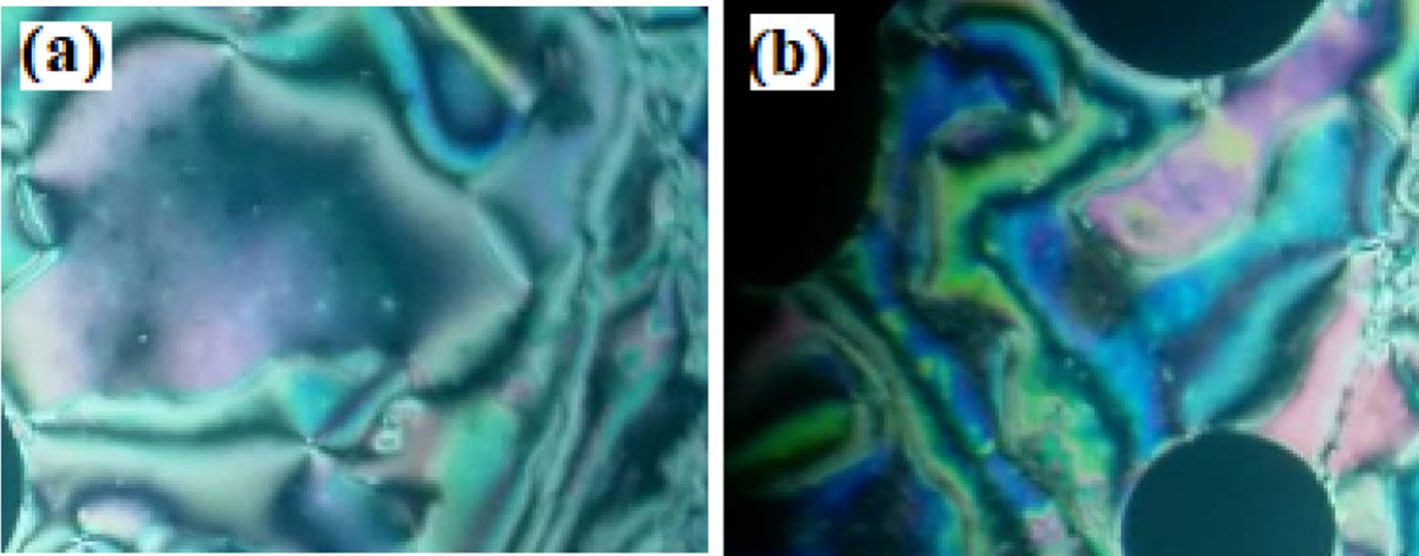
Nematic textures observed under POM for compound **I-6 **at (**a**) 160.0 °C and (**b**) 200.0 °C; the width of the images is about 40 µm.

**Figure 4:**
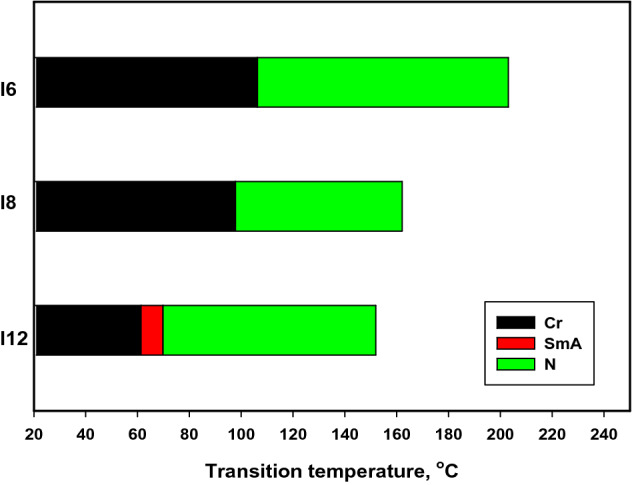
Impact of terminal chain-length on the mesomorphic transitions of the present homologue, **I-n**.

The mesomorphic and optical characteristics of the investigated synthesized derivatives have been analyzed by DSC and POM. Figure 2 displayed typical heating/cooling DSC thermograms of prepared compound **I-6** as a representative example. Figure 2 was observed that, the mesophase transitions from Cr → N, and N → I for short-chain length **I-6** derivative. Transition peaks observations vary according to the structural shape of synthesized materials, **I-n**. Significant endothermic and exothermic peaks were observed depending on the attached terminal alkoxy chain length group that is ascribed to mesomorphic transition and the cooling cycle confirmed those observed upon decrement the temperature. Optical images of the **I-6** derivative under POM are illustrated in Fig. 3. Schlieren/threads textures of the N mesophase were identified upon heating and cooling scans. The phase transition temperatures, as measured from DSC analysis, and their associated enthalpies for all the investigated compounds, **I-n**, are collected in Table 1. The effect of terminal alkoxy chain length on their mesomorphic behavior has been depicted in Fig. 4. Table 1 and Fig. 4 show that all prepared members of the series **I-n** are mesomorphic in nature with high mesomorphic thermal stability and a wide mesophase range dependent on their terminal chain length. Moreover, Compounds **I-6** and **I-8** are monomorphic possessing purely N phase while the longer chain compound **I-12** possesses two mesomorphic transitions (dimorphic) enantiotropically defined as SmA and N mesophases.

**Table 1:** Mesophase transition temperatures, ^o^C (enthalpy of transition), mesomorphic range (ΔT, ^o^C), and the normalized entropy of transition, ΔS/R, for present series **In**.

Comp	*T*_Cr-SmA_	*T*_Cr-N_	*T*_SmA-N_	*T*_N-I_	*ΔT*	*ΔS*_N-I_/R
**I-6**	–	106.3 (39.21)	–	203.1 (1.94)	96.8	0.49
**I-8**	–	97.8 (38.59)	–	162.1(1.69)	64.3	0.47
**I-12**	61.3 (35.27)	–	69.8 (3.21)	151.9 (1.75)	90.6	0.50

Cr–N = solid to the nematic mesophase transition.

Cr-SmA = solid to the smectic A mesophase transition.

SmA-N = smectic A to the nematic mesophase transition.

N-I = nematic to the isotropic liquid mesophase transition.

**ΔH**= enthalpy of transition, kJ/mole; ΔS/R= normalized entropy of transition, unitless (due to the entropy change ΔS is divided by R= gas constant).

It can also be seen from Table 1 and Fig. 4 that the melting point of compounds varies regularly with the chain length (n). Compound **I-6** exhibits an enantiotropic nematic phase with the highest nematic thermal stability and temperature range 203.1 and 96.8 °C, respectively. For **I-8** derivative, it has possesses also an enantiotropic N mesophase with nematogenic stability and range nearly 162.1 and 64.3 °C, respectively. While the derivative bearing the longest chain length (**I-12**) possesses less thermal nematic stability (151.9 °C) and the lowest melting temperature 61.3 °C. So that, compound **I-12** has induced smectic A mesophase and its mesomorphic range has been broader (90.6 °C). In general, the molecular architecture, polarizability, and dipole moment of the synthesized materials are highly impacted by the electronic nature of the terminals. In addition, the mesomorphic character is influenced by an increment in the polarity and/or polarizability of the molecular mesogenic moieties. The mesomorphic range of present investigated homologue increased in the order: **I-6** > **I-12** > **I-8**. The mesophase behavior of rod-like molecules is directly impacted by molecular–molecular interactions that depend essentially on their geometrical structure of the polar terminal and lateral groups and their special orientation. Mesomorphic properties observations results of the contribution of these factors to different extents. On the other hand, the DSC examination indicated to, the investigated imine derivatives exhibit high thermal stabilities more than 300 °C, which covers the transition window of mesophase temperature that detected thermally and extends over this transition too.

On the other hand, the normalized transition entropy changes, ΔS_N-I_/R, of the present series (**I-n**) are collected in Table 1. Data showed that small entropy changes values are observed that mainly depend on the kind of terminal substituents. The small values observed for the entropy change can be attributed to the decrease of the length-to-breadth ratio resulting from their lower anisotropy in terms of their molecular geometry^61–64^. The induction, conjugation forces, the specific dipolar interactions as well as the π–π stacking interactions^61–64^ play important roles in the molecular orientation and thus in the arrangement of molecules and formation of the mesophase. In addition, the thermal cis/trans isomerization of the azomethine linkage was an essential factor in the lower entropy changes observed, as documented in previous studies^65,66^. Moreover, due to their nematic nature, this of the mesophase, this was exhibits of the lowest order mesophase. While the higher entropy changes of SmA-N transition for compound **I-12** are attributed to the increment in its molecular biaxiality^67,68^.

### Comparison between the present investigated In series and their isomeric derivatives

**Figure 5:**
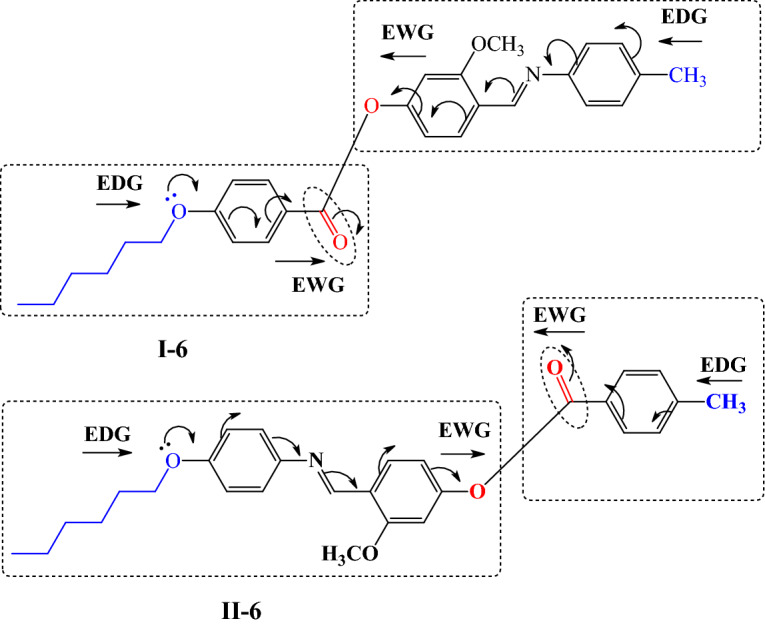
Schematic conjugation of compounds** I-6** and **II-6***;* Electron withdrawing group (EWG); Electron donating group (EDG).

In order to investigate the effect of exchange the mesogenic part between the aromatic rings on the mesophase and thermal behaviors of the compounds, thus a comparison is conducted between the presently prepared member **I-6** and their previously corresponding isomer, **II-6**^69^ for their mesophase behaviors. Compound **II-**6 possesses enantiotropic N mesophase with stability and temperature range nearly 141.3 and 51.1 °C, respectively. While the present investigated derivative **I-6** has a wide nematic range with high thermal stability. In addition, the conjugated Schiff's bases **I-6** and **II-6** (Fig. 5) suggests that the insertion of one more double bond stabilities of the mesophases and increment the phase transition temperatures. It seems that the increase in length of molecule contributes to these effects. The comparison revealed that the thermal stability of the formed mesophase varies according to the enhanced molecular dipole moment and polarizability of the mesogenic part, which is dependent upon the location of polar groups. Moreover, the mesophase range and stability depend on the location of the terminal and linking groups in the mesogenic skeleton of the molecule.
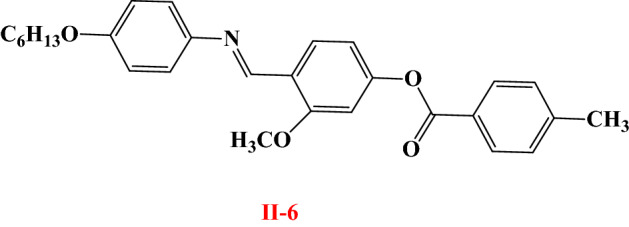

### Optical spectra and energy gap calculation

**Figure 6:**
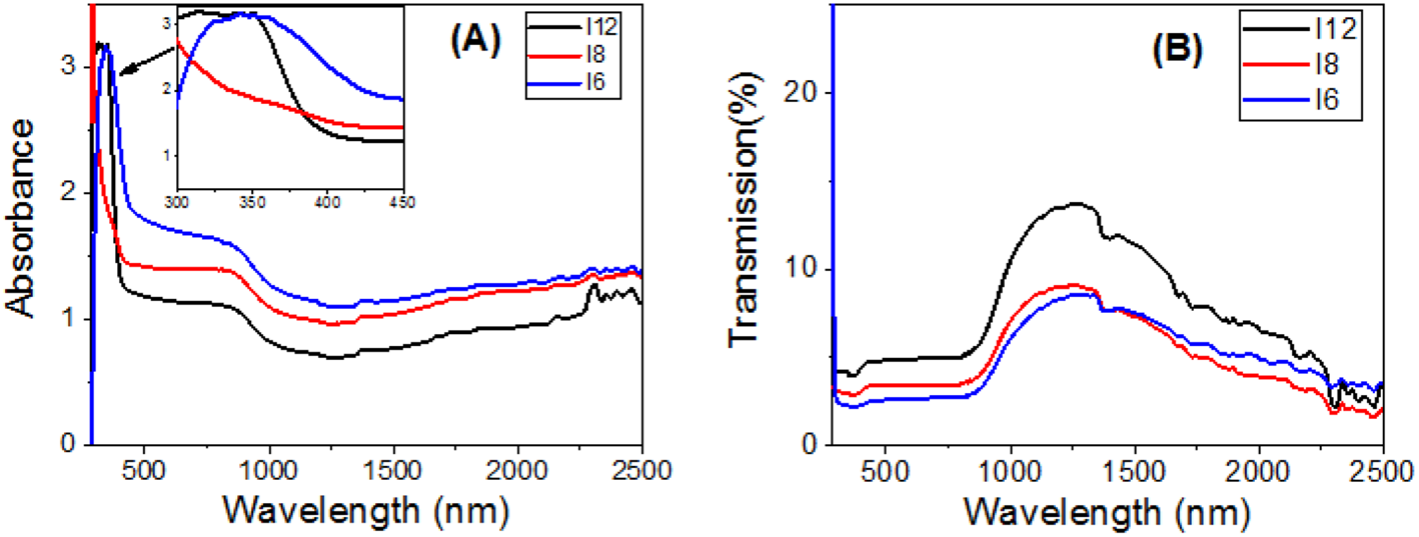
Effective optical (**A**) absorbance and (**B**) transmittance spectra of **I-6, I-8, and I-12** in liquid crystal phase designed at 140 °C.

Because liquid crystals are anisotropic materials and mesogenic materials cannot be aligned using existing techniques, the measured physical properties are referred to as effective optical absorbance and effective electrical conductivity. A Perkin Elmer spectrophotometer (Lambda 950 UV–VIS–NIR) was used to measure the effective optical absorbance and transmission spectra of the present investigated series, **I-n**, over a wavelength range of 250 to 2500 nm utilizing a blank glass substrate in the reference beam. The samples were sandwiched between two glass substrates. Figure 6A,B shows how the effective absorbance and transmittance spectra of the films are affected by wavelength. In comparison to **I8** and **I12**, the effective absorbance spectra in Fig. 6A show that **I-6** has a high absorption behavior. For present homologue **I-n, **all films display high absorbance up to 402, 416, and 450 nm for **I-6**, **I-8,** and **I-12**, respectively***.*** The absorbance then drops to a plateau at about 850 nm, before dropping again to a minimum absorbance around 1268 nm. Figure 6A shows a strong absorption band for **I-6** at 342 nm, which is blue-shifted by increasing the terminal length of a flexible chain of prepared series for **I-8** and **I-12**. The absorbance intensity is in the order **I-6** > **I-8** > **I-12**. The right edge of the absorption band is red-shifted leading to an increase in the full width at half maximum. This red-shift is mainly attributed to the size effects, where small size reduces spin–orbit coupling and moderates the exciton positions^70^. This red-shift and high absorption in UV and visible regions is a desirable feature for design energy-efficient solar cells^71^. The optical spectra refer to the homogeneity of the prepared films by decreasing the terminal length of the prepared series because the optical properties depend mainly on morphology and chemical composition. All films showed transmission less than 5% in the wavelength range from 300 to 800 nm, Fig. 6B. Then, the transmission increased exponentially in the near IR region to reach maxima of ~ 14%, 9%, and 8%@1266 nm for **I-12**, **I-8**, and **I-6**, respectively. After that, the transmission decreased as the wavelength increased.

**Figure 7:**
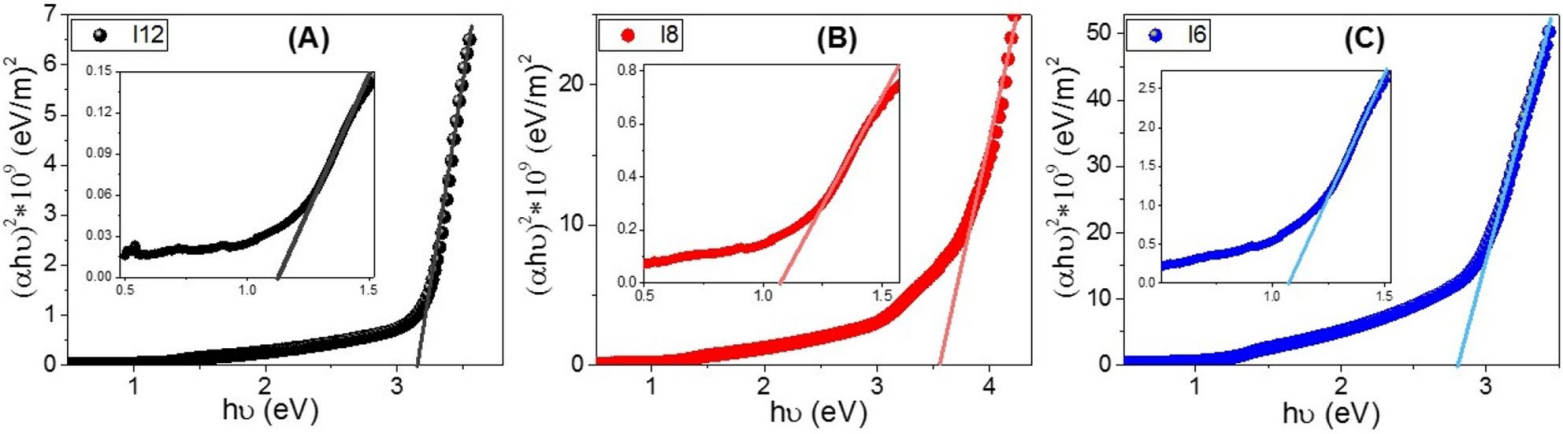
Calculation of energy gap for (**A**) **I-6,** (**B**) **I-8**, and (**C**) **I-12** in liquid crystal phase designed at 140 °C.

The concept of the crystalline solids band gap (*E*_*g*_) can be expanded to include disordered and highly deficient phases, such as amorphous bodies (glass) and liquids. In this scenario, Eg can be called quasigap (Eg*), which refers to the existance of both localized and widespread electronic states near the edges, as well as in its depth^72^. Pathak et al. calculated the optical band gaps of TiO_2_ dispersed nematic liquid crystals with the help of Tauc plot^73^ According to Tauc equation and because our samples are almost measured in solid phase, the energy gap (*E*_*g*_) was calculated at 20 °C by Eq. (1) ^74^:

1 $$(\alpha_{a} h\nu )^{{2}} = {\text{ A}}(h\nu - \, E_{g} )$$

where *hν* is the photon energy and *α*_*a*_ is the absorption coefficient*.* The values of *E*_*g*_ for **I-6**, **I-8,** and **I-12** are obtained by extending the linear segments of the plot of (*α*_*a*_ *hυ*)^2^ vs. *hυ* to *zero* as shown in Fig. 7A–C. Interestingly as reported in Table 2, there are two values of the band gaps for the **I-6**, **I-8**, and **I-12**. For the shortest terminal chain compound (**I-6),** its values of the band gaps are 1.07 and 2.79 eV, which are suitable for solar energy applications^10–14^. By increasing the terminal length of the flexible chain, the values of the band gaps are shifted to 1.13 and 3.14 eV for the **I-12 **derivative.

**Table 2:** Values of the energy gap, Eg, and Urbach energy, *E*_*U*_, of **I-6, I-8,** and **I-12** derivatives in liquid crystal phase designed at 140 °C.

**C**ompound	Eg (eV)	Eu (eV) ± SD	R^2^
**I-12**	1.13	0.956 ± 0.010	0.9988
	3.14	0.629 ± 0.040	0.9984
**I-8**	1.09	0.634 ± 0.006	0.9984
	3.55	0.539 ± 0.007	0.9985
**I-6**	1.07	0.857 ± 0.009	0.9988
	2.79	0.990 ± 0.009	0.9994

The observed increase in the main bandgap from 2.79 eV for I6 to 3.14 eV for **I**12 is ascribed to the influence of the density of localized states. This behavior is consistent with the previously reported studies^75^. The reduction of the bandgap is very important for solar energy applications, specially photoelectrochemical hydrogen generation, and solar cells^76–78^.

**Figure 8:**
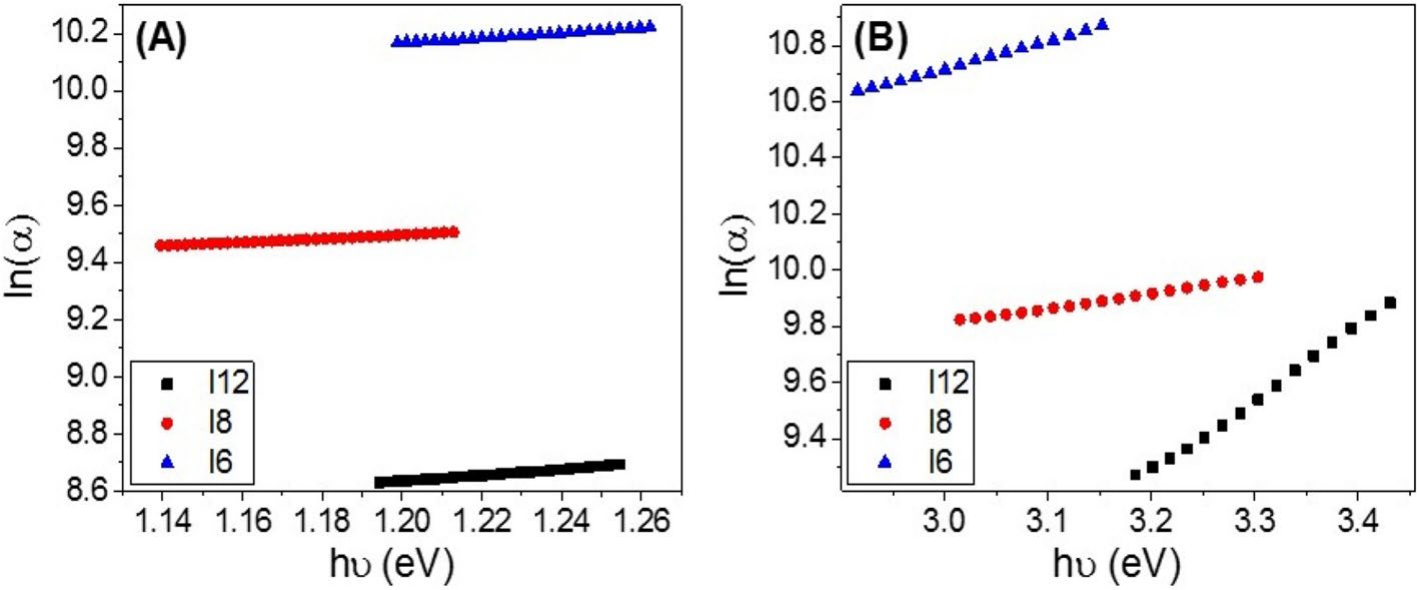
Calculation of Urbach energy (**A**) Eu_1_ and (**B**) Eu_2_ for **I-6, I-8**, and **I-12** in liquid crystal phase designed at 140 °C.

Urbach energy (*E*_*U*_) refers to the disorder in the material and represents the width of the exponential absorption edge (Urbach tail of the valence and conduction bands^79^. I.e., Urbach energy is the energy that refers to the creation of localized energy states at the boundaries of the energy gap due to structural disorder of the material and gives the spectral dependence of the absorption coefficients at photon energies less than the bandgap of the material. The exponential dependency of the *E*_*U*_ can be determined according to the following equation^79^:

2 $$\alpha_{a} = \alpha_{ao} {\text{exp }}\left( {E_{ph} /E_{u} } \right) \to E_{u} = \, \delta E_{ph} /\delta (ln(\alpha_{a} ))$$

where *α*_*ao*_ is the band tail parameter that can be given by^80^:

3 $$\alpha_{ao} = (4\pi \, \sigma_{o} \, / \, x \, \Delta E \, c \, )^{1/2}$$

where *c* is the speed of light, *σ*_*o*_ is electrical conductivity at absolute zero, *ΔE* represents the width of the tail of the localized state in the forbidden gap. Figure 8A and B shows the plot of ln(*α*) vs. *hν* for the two band gaps of **I-6, I-8**, and **I-12**. The values of *E*_*U1*_ and *E*_*U2*_ were obtained from the slopes of the linear fitting of these curves and reported in Table 2. The statistical parameters, standard deviation (SD) and correlation coefficient (R^2^), are also reported in this table. The values are 0.857 ± 0.009 and 0.990 ± 0.009 eV for **I-6** and 0.956 ± 0.010 and 0.629 ± 0.040 eV for **I-12**, which refers to the extension of the bandgap edges to cover a wide range of the spectral range.

### Electrical properties

**Figure 9:**
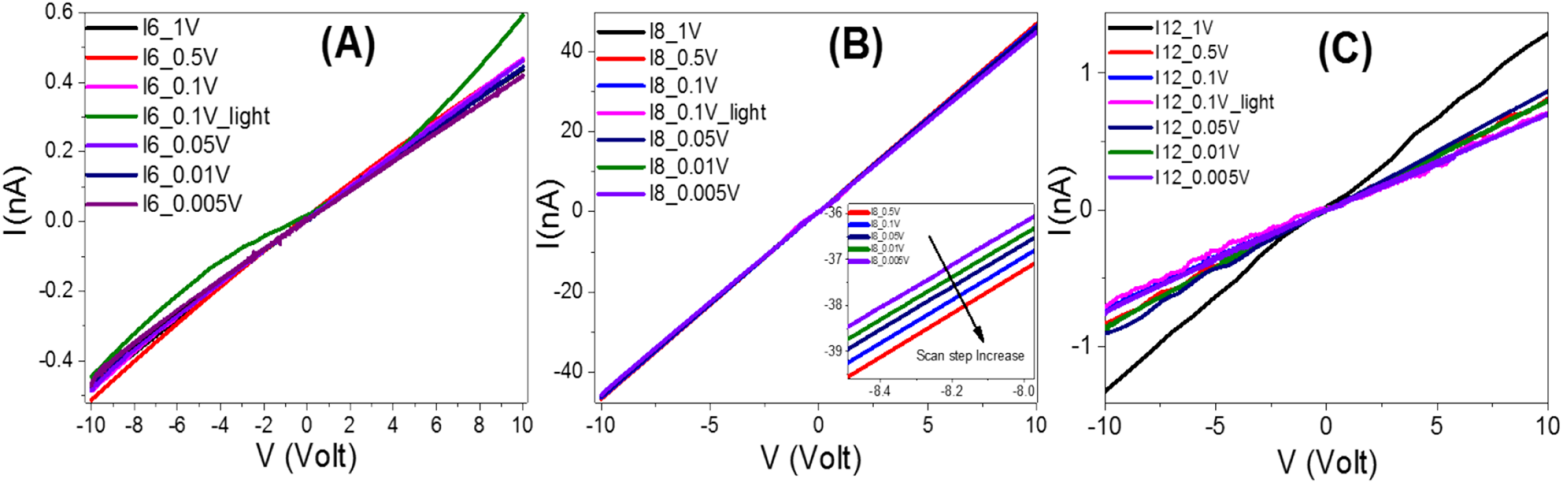
Electrical voltage-current characteristics of (**A**) **I-6**, (**B**) **I-10**, and (**C**) **I-12** samples in liquid crystal phase designed at 140 °C.

**Figure 10:**
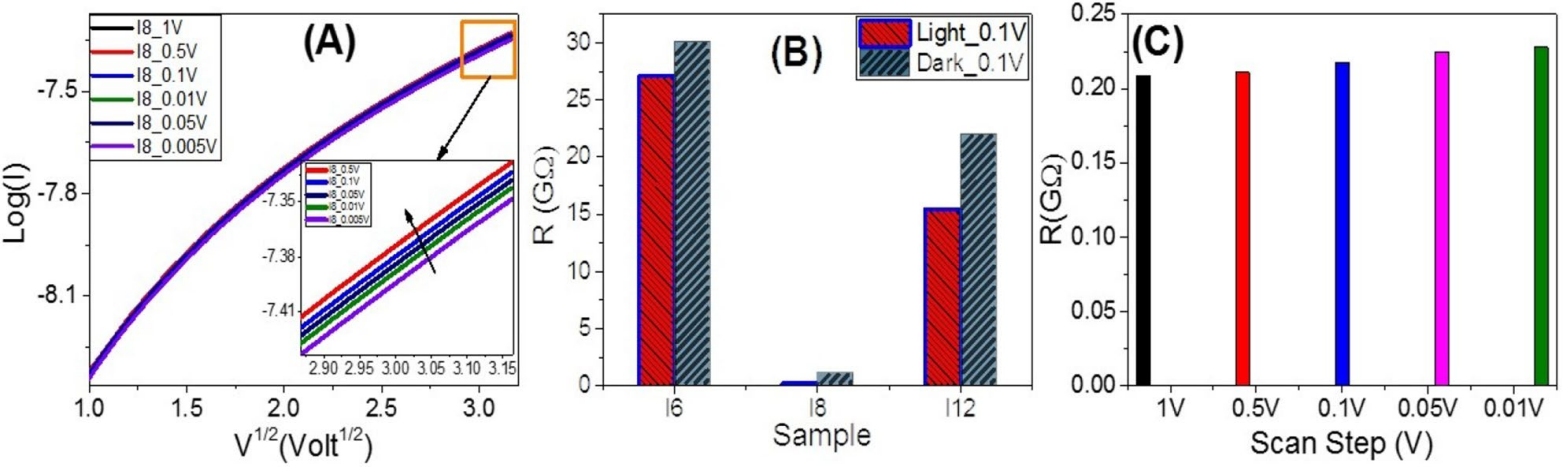
(**A**) Log(I) vs. V^0.5^ For **I-8** sample at different step scan; (**B**) effective electric resistance in dark and white light illumination conditions for **I-6, I-8**, and **I-12** samples in liquid crystal phase designed at 140 °C; and (**C**) effective electric resistance at different scan step for sample **I-8**.

The effective electrical properties of the investigated films are tested using a Keithley measurement source unit (Model 4200 SMU). The samples were provided with Ohmic contacts using silver paste (Resistivity < 0.04 Ω.cm). Variation of the applied voltage (V) from − 10 to 10 V with different scan steps, 1V to 0.005V, is used to record the current-voltage (*I–V*) characteristics of the **I-6**, **I-8**, and **I-12** films, as shown in Fig. 9A–C. The behaviors are almost linear (Ohmic behaviors). As a result, the materials' resistances are nearly constant and independent of the current flowing through them. Recent research has discovered that at low voltage, polymeric and organic systems behave like Schottky diodes. The Schottky diode is a semiconductor diode made up of a semiconductor and a metal junction that has a low forward voltage drop and a fast switching operation. But in the present investigation, the relation between log (I) and V^1/2^ is non-linear as illustrated in Fig. 10A, which implies that our films do not follow the Schottky diode behavior. Under white light illumination, Fig. 9A, the values of the current increased, and the I-V behavior is shifted from the ohmic relation. As the scan step increased, the current intensity is also increased, insets of Figs. 9B and 10A. Figure 10B shows the obtained values of electric resistance for **I-6**, **I-8**, and **I-12** in dark and under white light illumination. The resistances of the films are in the order R_***I6***_ > R_***I12***_ > R_***I8***_. The values of the electrical resistances are 30.06, 1.10, and 21.99 GΩ in dark, which are decreased to 27.14, 0.22, and 15.45 GΩ under white light illumination for **I-6**, **I-8**, and **I-12**, respectively. The observed resistance behavior may be attributed to the mesomorphic range (*ΔT*, Table 1)of prepared sample where the liquid crystalline phase range increasing in order **I-6** > **I-12** > **I-8**. The mesomorphic range of liquid crystalline materials is affected by many parameters as the polarity and polarizability of whole molecular shape this would be reflected in the resulting resistance data. The observation of more ordered smectic phase for the longer chain length derivative (**I-12**) could be explained in the term of the enhancement of the polarity and the polarizability with lengthening of the alkoxy terminal chain ^32,33^. The effective electric resistance of **I-8** film is increased from 20.8 GΩ to 22.8 GΩ by decreasing the scan step from 1 V to 0.01 V as shown in Fig.10C which has the lowest entropy change value (see Table 1). The values of the effective electric conductance (σ) are obtained and shown in Fig. S1 (Supplementary Data). The value of the effective electrical conductance is increased from 0.91 nS in dark to 4.60 nS under white light illumination since the electrical conductance depends mainly on the number and mobility of charge carriers^81,82^. This indicates the coherent photocurrent generation, which is the basis of the photovoltaic cell^83^.

## Conclusion

New mesomorphic laterally methoxy-substituent homologues series named, (*E*)-3-methoxy-4-[(*p*-tolylimino)methyl]phenyl 4-alkoxybenzoate (**I-n**), were synthesized and characterized by different thermal, optical, and electrical tools. The prepared series included three materials that differ from each other by the terminal length of the flexible chain. A lateral OCH_3_ group is inserted into the central benzene ring. Elucidations of structures were carried out by elemental analyses, FT-IR, and NMR spectroscopy. Characterizations of present compounds are investigated using DSC, POM, UV spectrophotometer, Keithley measurement-source unit, and UV/Vis/IR Perkin Elmer spectrophotometer. DSC and POM investigations indicate that all synthesized compounds are enantiotropic monomorphic exhibiting only N mesophase, except for the longest chain derivative (**I-12**) that is dimorphic possesses smectic A and N phases. Additionally, all compounds have a broad mesomorphic range with high thermal nematic stability. A comparative study was made between the present derivative (**I-6**) and their corresponding isomer and results indicated that the exchange of the location of the polar CH_3_ group influences the N mesophase range and stability. The effective electrical measurements revealed that, with electric resistances in the GIGA range, all investigated derivatives (**I-n**) exhibited Ohmic behaviors. The effective electric conductivity of the compound **I-8** is five times higher under white light illumination than in dark conditions. Moreover, In the UV and visible light ranges, this member (**I-8**) has revealed two direct optical band energy gaps. Further, it was found that the band energy gaps for **I-6** are 1.07 eV and 2.79 eV, which confirming that it is appropriate for solar energy applications.

## Supplementary Information﻿

Supplementary Information.
