# Author Correction: Optical investigations and photoactive solar energy applications of new synthesized Schiff base liquid crystal derivatives

**DOI:** 10.1038/s41598-021-97264-w

**Published:** 2021-08-31

**Authors:** Fowzia S. Alamro, Sobhi M. Gomha, Mohamed Shaban, Abeer S. Altowyan, Tariq Z. Abolibda, Hoda A. Ahmed

**Affiliations:** 1 Department of Chemistry, College of Science, Princess Nourah bint Abdulrahman University, Riyadh, 11671 Saudi Arabia; 2 Department of Chemistry, Faculty of Science, Cairo University, Cairo, 12613 Egypt; 3 Chemistry Department, Faculty of Science, Islamic University in Almadinah Almonawara, Almadinah Almonawara, 42351 Saudi Arabia; 4 Nanophotonics and Applications Labs, Department of Physics, Faculty of Science, Beni-Suef University, Beni-Suef, 62514 Egypt; 5 Department of Physics, Faculty of Science, Islamic University in Almadinah Almonawara, Almadinah Almonawara, 42351 Saudi Arabia; 6 Department of Physics, College of Science, Princess Nourah bint Abdulrahman University, Riyadh, 11671 Saudi Arabia; 7 Chemistry Department, College of Sciences, Taibah University, Yanbu, 30799 Saudi Arabia

Correction to: *Scientific Reports* https://doi.org/10.1038/s41598-021-94533-6, published online 22 July 2021

In the original version of this Article, Tariq Z. Abolibda was incorrectly affiliated with ‘Nanophotonics and Applications Labs, Department of Physics, Faculty of Science, Beni-Suef University, Beni-Suef, 62514, Egypt’. The correct affiliation is listed below.

Chemistry Department, Faculty of Science, Islamic University in Almadinah Almonawara, Almadinah Almonawara, 42351, Saudi Arabia

In addition, in Affiliations 1 and 6,

“Princess Nourah Bint Abdulrahman University”

now reads:

“Princess Nourah bint Abdulrahman University”

The original Article and accompanying Supplementary Information file have been corrected.

