# Supplementary material for: Optical investigations and photoactive solar energy applications of new synthesized Schiff base liquid crystal derivatives
Source: Sci Rep. 2021 Jul 22;11:15046. doi: 10.1038/s41598-021-94533-6 (PMC8298406; doi:10.1038/s41598-021-94533-6)
Supplement: Supplementary file 1 — Supplementary Information. [file 41598_2021_94533_MOESM1_ESM.docx]

**Supplementary Materials**

**Optical investigations and Photoactive solar Energy applications of New synthesized Schiff base liquid crystal derivatives**

Fowzia S. Alamro^1^, Sobhi M. Gomha^2,3^, Mohamed Shaban^4,5^, Abeer S. Altowyan^6^ ,Tariq Z. Abolibda^3^ , and Hoda A. Ahmed^2,7^*

*^1^Department of Chemistry, College of Science, Princess Nourah bint Abdulrahman University, Riyadh 11671, Saudi Arabia,* *fsalamro@pnu.edu.sa*

*^2^Department of Chemistry, Faculty of Science, Cairo University, Cairo 12613, Egypt,* [*ahoda@sci.cu.edu.eg*](mailto:ahoda@sci.cu.edu.eg) *(HA.A),* [*sm.gomha@iu.edu.sa*](mailto:sm.gomha@iu.edu.sa) *(SM.G)*

*^3^Chemistry Department, Faculty of Science, Islamic University in Almadinah Almonawara, Almadinah Almonawara, 42351, Saudi Arabia,* [*t.z.a@iu.edu.sa*](mailto:t.z.a@iu.edu.sa)*.*

*^4^Nanophotonics and Applications Labs, Department of Physics, Faculty of Science, Beni-Suef University, Beni-Suef 62514, Egypt,* [*mssfadel@aucegypt.edu*](mailto:mssfadel@aucegypt.edu)

*^5^Department of Physics, Faculty of Science, Islamic University in Almadinah Almonawara, Almadinah Almonawara, 42351, Saudi Arabia*

*^6^Department of physics, College of Science, Princess Nourah bint Abdulrahman University, Riyadh 11671, Saudi Arabia*

*^7^Chemistry Department, College of Sciences, Yanbu, Taibah University, Yanbu 30799, Saudi Arabia*

** Correspondence: Hoda A. Ahmed,* [*ahoda@sci.cu.edu.eg*](mailto:ahoda@sci.cu.edu.eg)

1. ***Materials***

4-Hexyloxybenzoic acid, 4-octoyloxybenzoic acid, 4-dodecyloxybenzoic acid 4-hydroxy-2-methoxy benzaldehyde, and 4-methyle aniline, were purchased from Sigma Aldrich (Germany). dichloromethane, *N,N'*-dicyclohexylcarbodiimide (DCC), ethanol and 4-dimethylaminopyridine (DMAP) were purchased from Aldrich (Wisconsin, USA).

1. *Synthesis of (E)-3-methoxy-4-[(p-tolylimino)methyl]phenol (****3****)*

A mixture of 4−hydroxy-2-methoxybenzaldehyde (1.52g, 10 mmol) and 4-toluidine (1.07g, 10 mmol) in ethanol (20 mL) were refluxed for two hours (monitored by TLC). The mixture was cooled to room temperature and filtered. The obtained solid was washed was cold ethanol and recrystallized twice from hot ethanol to give pure imine compound **3** as indicated by TLC analysis. The melting points and IR data determined of the prepared imine **3:** mp 124-126^°^C (Lit mp 123-124^°^C) [i].

1. *Synthesis of* (*E*)-3-methoxy-4-[(*p*-tolylimino)methyl]phenyl 4-alkloxybenzoates (**I-n**)

A mixture of imine compound **3** (2.41g, 10 mmol) and the appropriate 4-alkoxy benzoic acid derivatives **4** (10 mmol for each) in dry methylene chloride (25 mL) containing *N*,*N′−*dicyclohexylcarbodiimide (DCC, 10 mmol) and few crystals of 4*-*dimethylaminopyridine (DMAP), as catalyst, were left to stand for 72 hours at room temperature with continuous stirring. The solid separated was then filtered off and the solution evaporated. The solid residue obtained was recrystallized from ethanol to give TLC pure products. The purity of the prepared samples was checked with thin-layer chromatography (TLC) using TLC sheets coated with silica gel (E Merck), and CH_2_Cl_2_/CH_3_OH (9:1) as eluent, whereby only one spot was detected by a UV-lamp. Infrared spectra (IR), ^1^H-NMR, and elemental analyses for compounds investigated were consistent with the structures assigned.

The physical analyses data of products **I-n** are listed below:

**(*E*)-3-Methoxy-4-[(*p*-tolylimino)methyl]phenyl 4-hexyloxybenzoate,** **I*-*6:**

Yield: 91.9%; mp 106.0 ^°^C, FTIR (ύ, cm^−1^): 3034, 2923 (C-H), 1728 (C=O), 1613 (C=N). ^1^H-NMR (400 MHz, DMSO): *δ*/ppm: 0.91 (t, 3H, CH_3_(CH_2_)_3_CH_2_CH_2_O-), 1.23-1.70 (m, 6H, CH_3_(CH_2_)_3_CH_2_CH_2_O-), 1.76-178 (m, 2H, CH_3_(CH_2_)_3_CH_2_CH_2_O-), 2.45 (s, 3H, Ar-CH_3_), 3.79 (s, 3H, OCH_3_), 4.02-4.07 (t, 2H, CH_3_(CH_2_)_3_CH_2_CH_2_O-), 6.56-6.58 (d, 2H, Ar−H), 7.03-7.05(d, 1H, Ar−H), 7.23-7.30 (d, 2H, Ar−H), 7.41-7.45 (d, 2H, Ar−H), 7.69 (s, 1H, Ar−H), 8.02-8.07 (m, 3H, Ar−H), 8.60 (s, 1H, CH=N); ^13^C-NMR (400 MHz, DMSO): *δ*/ppm: 13.90, 22.17 (CH_3_), 24.08, 25.31, 28.72, 34.15 (CH_2_), 55.95(OCH_3_), 68.05 (CH_2_-O), 106.67, 111.37, 114.51, 120.03, 121.94, 123.57, 129.17, 131.69, 132.18, 134.78, 138.91, 142.15, 150.01 (Ar-C), 153.88 (C=N), 160.49 (Ar-C-OR), 163.26 (C=O). Anal. Calcd. for C_28_H_31_NO_4_ (445.55): C, 75.48; H, 7.01; N, 3.14. Found: C, 75.30; H, 6.93; N, 3.05%.

**(*E*)-3-Methoxy-4-[(*p*-tolylimino)methyl]phenyl 4-octyloxybenzoate, I-8**:

Yield: 93.7%; mp 97.0 ^°^C, FTIR (ύ, cm^−1^): 3051, 2922 (C-H), 1723 (C=O), 1607 (C=N). ^1^H-NMR (400 MHz, DMSO): *δ*/ppm: 0.82 (t, 3H, CH_3_(CH_2_)_5_CH_2_CH_2_O-), 1.20-1.70 (m, 10H, CH_3_(CH_2_)_5_CH_2_CH_2_O-), 1.76-178 (m, 2H, CH_3_(CH_2_)_5_CH_2_CH_2_O-), 2.45 (s, 3H, Ar-CH_3_), 3.79 (s, 3H, OCH_3_), 4.04-4.06 (t, 2H, CH_3_(CH_2_)_5_CH_2_CH_2_O-), 6.53-6.54 (d, 2H, Ar−H), 7.05-7.07 (d, 1H, Ar−H), 7.26-7.28 (d, 2H, Ar−H), 7.42-7.44 (d, 2H, Ar−H), 7.69 (s, 1H, Ar−H), 8.01-8.05 (m, 3H, Ar−H), 8.60 (s, 1H, CH=N); ^13^C-NMR (400 MHz, DMSO): *δ*/ppm: 13.95, 22.10 (CH_3_), 24.03, 25.33, 25.44, 28.72, 31.25, 34.50 (CH_2_), 55.91(OCH_3_), 68.01 (CH_2_-O), 106.69, 111.27, 114.73, 120.29, 122.56, 123.57, 129.17, 130.31, 132.12, 134.78, 138.99, 142.29, 150.09 (Ar-C), 153.96 (C=N), 160.76 (Ar-C-OR), 163.29 (C=O). Anal. Calcd. for C_30_H_35_NO_4_ (473.60): C, 76.08; H, 7.45; N, 2.96. Found: C, 75.92; H, 7.31; N, 2.77%.

**(*E*)-3-Methoxy-4-[(*p*-tolylimino)methyl]phenyl 4-dodecyloxybenzoate,**  **I-12**:

Yield: 92.0%; mp 61.0^°^C, FTIR (ύ, cm^−1^): 3028, 2925 (C-H), 1730 (C=O), 1611 (C=N). ^1^H-NMR (400 MHz, DMSO): *δ*/ppm: 0.80 (t, 3H, CH_3_(CH_2_)_9_CH_2_CH_2_O-), 0.96-1.68 (m, 18H, CH_3_(CH_2_)_9_CH_2_CH_2_O-), 1.76-178 (m, 2H, CH_3_(CH_2_)_5_CH_2_CH_2_O-), 2.45 (s, 3H, Ar-CH_3_), 3.79 (s, 3H, OCH_3_), 4.03-4.06 (t, 2H, CH_3_(CH_2_)_9_CH_2_CH_2_O-), 6.53-6.54 (d, 2H, Ar−H), 7.06-7.09 (d, 1H, Ar−H), 7.26-7.34 (d, 2H, Ar−H), 7.43-7.44 (d, 2H, Ar−H), 7.69 (s, 1H, Ar−H), 8.01-8.05 (m, 3H, Ar−H), 8.60 (s, 1H, CH=N); ^13^C-NMR (400 MHz, DMSO): *δ*/ppm: 13.95, 22.10 (CH_3_), 24.01, 24.45, 24.94, 25.31, 25.40 28.69, 28.94, 31.29, 33.33, 34.48 (CH_2_), 55.92 (OCH_3_), 68.00 (CH_2_-O), 106.68, 111.27, 114.73, 122.56, 122.87, 123.58, 129.17, 130.28, 132.11, 138.98, 142.28, 149.31, 153.92 (Ar-C), 156.61 (C=N), 160.78 (Ar-C-OR), 163.44 (C=O). Anal. Calcd. for C_34_H_43_NO_4_ (529.71): C, 77.09; H, 8.18; N, 2.64. Found: C, 77.16; H, 8.07; N, 2.59%.

1. ***Characterization***

Perkin-Elmer B25 (Perkin-Elmer, Inc., Shelton, CT USA) spectrophotometer was used for infrared spectra measurements. Varian EM 350L 500 MHz spectrometer (Oxford, UK) was used for recording ^1^HNMR spectra using tetramethyl silane as internal standard in CDCl_3_; the chemical shift values recorded as δ (in ppm units). Thermo Scientific Flash 2000 CHS/O Elemental Analyzer, Milan, Italy was used for Elemental analyses.

TA Instruments Co. (Q20 Differential Scanning Calorimeter, DSC; USA) was used for recording phase transitions. DSC calibration was carried out using lead and indium to calibrate the melting temperatures and enthalpies. Samples of 2–3 mg were used in aluminum pans for DSC investigation. The heating rate was 10°C/min in nitrogen gas as an inert atmosphere (30 ml/min). All transitions temperatures were measured form the second heating scan.

Transition temperatures for the prepared compounds were checked and phases identified by Polarized optical microscope (POM, Wild, Germany) attached with Mettler FP82HT hot stage.

**
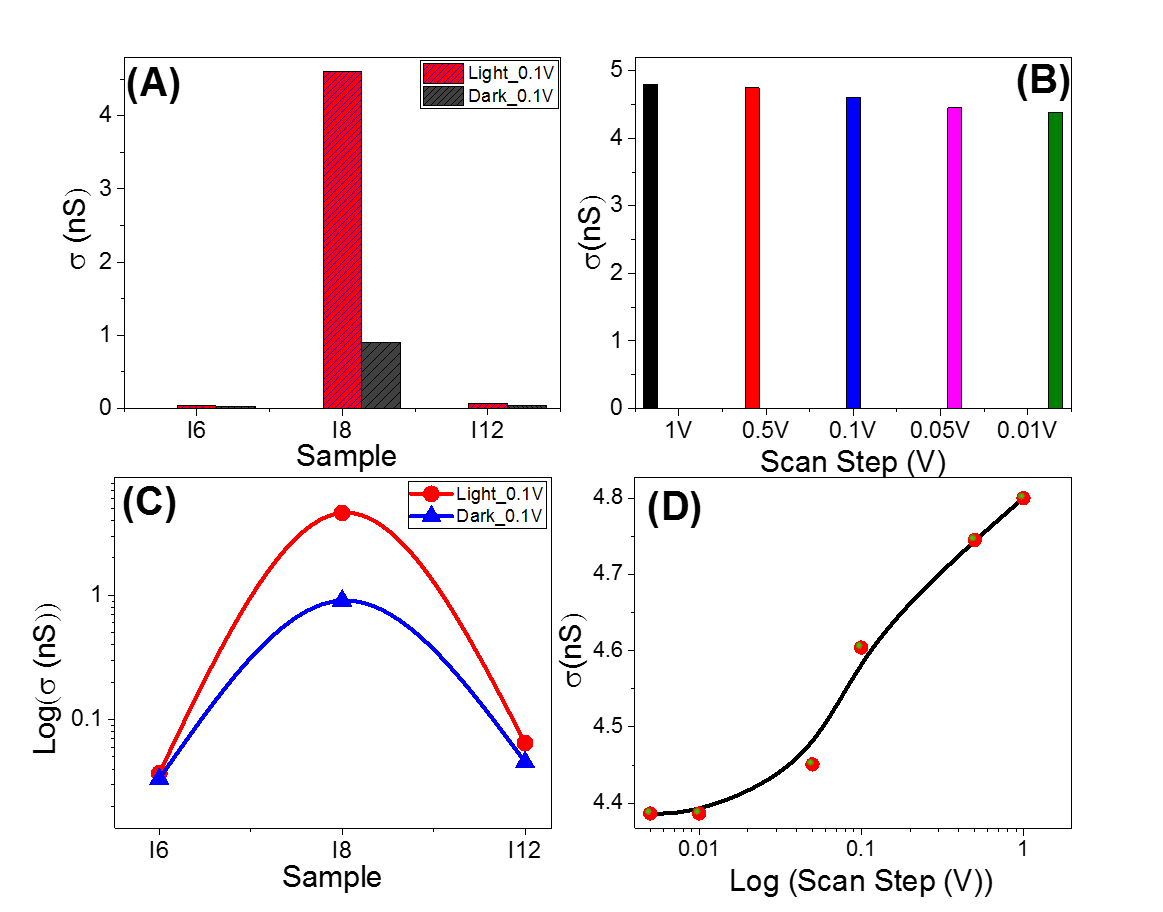
**

**Figure S1**. (A,C) Effective electric conductance in dark and white light illumination conditions for **I-6, I-8**, and **I-12** films; and (B,D) Effective electric conductance at different scan steps for sample **I-8**. A Semi-log scale is used in (C and D) to shows the difference between the samples and the dependence of the behavior of **I-8** on the scan rate.

**References:**

i. M. Al-Mamary, S.I. Abdelwahab, H. M. Ali, S. Ismail, M. A. Abdulla, P. Darvish, Asian Journal of Chemistry 24 (2012) 4335-4339.
